# The Effect of Pterygomasseteric Sling's Area in the Postoperative Stability after Mandibular Setback Surgery

**DOI:** 10.1155/2017/7216120

**Published:** 2017-10-09

**Authors:** Chun-Ming Chen, Chun-Chan Ting, Jung-Hsuan Cheng, Kun-Jung Hsu, Yu-Chuan Tseng

**Affiliations:** ^1^Graduate Institute of Dental Sciences, School of Dental Medicine, Kaohsiung Medical University, Kaohsiung, Taiwan; ^2^Department of Oral and Maxillofacial Surgery, Kaohsiung Medical University Hospital, Kaohsiung Medical University, Kaohsiung, Taiwan; ^3^Department of Restorative Dentistry and Endodontology, Graduate School of Medical and Dental Sciences, Kagoshima University, Kagoshima, Japan; ^4^Department of Orthodontics, Kaohsiung Medical University Hospital, Kaohsiung, Taiwan; ^5^Kaohsiung Municipal Ta-Tung Hospital, Kaohsiung, Taiwan

## Abstract

**Purpose:**

The purpose of the present study was to investigate the correlation between the postoperative stability and area of pterygomasseteric sling (PMS).

**Materials and Methods:**

Forty patients of mandibular prognathism were treated by isolated mandibular setback. Serial lateral cephalograms were collected (preoperatively [T1], immediately after surgery [T2], and more than 1 year postoperatively [T3]). The postoperative stability (T32) was divided into 3 groups (total, forward, and backward movements). The areas of PMS, immediate surgical changes (T21), postoperative stability (T32), and final surgical change (T31) were analyzed by Student's* t*-test, Pearson's correlation coefficient, and multiple linear regression analysis.

**Results:**

The amount of mean setback (T21) was 12.6 mm in total group, 13.8 mm in forward group, and 10.8 mm in backward group. In the total group, postoperative stability (T32) was 0.6 mm forward and reduction area of PMS (T31) was 291 mm^2^ (17.2%). The reduction area of PMS (T31) was 298.2 mm^2^ (18%) and 263.1 (15.3%) mm^2^ in the forward group (3 mm) and backward group (2.4 mm), respectively. However, reduction area of PMS (T31) showed weak correlation with postoperative stability (T32) in all groups.

**Conclusion:**

Total and forward groups presented significant correlations between postoperative stability (T32) and amount of setback (T21).

## 1. Introduction

Mandibular prognathism is induced by hypergenesis and results in a subsequent excess of the mandible, which leads to obvious protrusion of the chin area and lower lip, as well as depressed morphology in the facial form. The harmony of the facial form is therefore ruined, and the psychology of the patient is affected. On the effects of inheritance on the facial bone development of humans, similar facial characteristics are frequently seen among brothers, sisters, and parents in the families of patients with mandibular prognathism. Hunter et al. [1] reported that the size of all the facial bones of parents and their children among the 38 families studied was strongly correlated to inheritance. The genetic correlation between fathers and children was significantly higher than that between mothers and children.

Slight mandibular prognathism can be corrected by orthodontic treatment. For the treatment of developing children, orthopedic devices can be added as supplement of orthodontic procedures. However, orthognathic surgery is needed and combined orthodontic treatment for the correction of patients with moderate to severe conditions. Orthognathic surgery for the treatment of mandibular prognathism has been improved in many different ways from the old extraoral approach to the current intraoral approach. Sagittal split ramus osteotomy (SSRO) and intraoral vertical ramus osteotomy (IVRO) are the most common methods currently used for treatment of mandibular prognathism. Both of the procedures mentioned above have been reported with postoperative relapse [2–7]. Regardless of the kind of surgery performed to treat mandibular prognathism, osteotomy position of the mandible could affect the postoperative stability between the proximal and distal segments. In addition, the process of bone healing between the proximal and distal segments is closely related to the level of increase in activities and amount of force applied by the pterygomasseteric sling (PMS). The PMS [8] is composed of the masseter and medial pterygoid muscles. The insertion joins the both muscles to form a common tendinous sling that allows the medial pterygoid and masseter to be powerful elevators of the jaw.

Since PMS could be stretched by mandibular setback procedure, the area changes of the PMS's reattachment to the ramus maybe play an important remodeling role for influencing postoperative stability. Until now, there are no reports concerning the PMS's area changes by remodeling after mandibular setback surgery. The purpose of this study is to investigate the correlation between the postoperative changes of PMS and mandibular stability in treatment of mandibular prognathism by IVRO.

## 2. Materials and Methods

Forty patients needing surgical correction of mandibular prognathism were treated at the Department of Oral and Maxillofacial Surgery of the Kaohsiung Medical University Hospital. The inclusion criteria were as follows: (1) patients who had mandibular prognathism; (2) patients with no craniofacial anomalies; (3) patients with no history of trauma or recognized syndromes; (4) patients with no facial growth at the time of operation, which was verified by serial cephalometric records and measurements; (5) patients who had received preoperative and postoperative orthodontic treatments; (6) patients who were operated by a single jaw procedure. The surgical technique was used the IVRO method for mandibular setback procedure. The IVRO method which bone cuts are continued from superior to the sigmoid notch, behind the mandibular foramen, and inferior to the gonial angle. All patients had no fixation between the proximal and distal segments. Postoperatively, all patients maintained intermaxillary fixation for 6 weeks.

**Figure 1:**
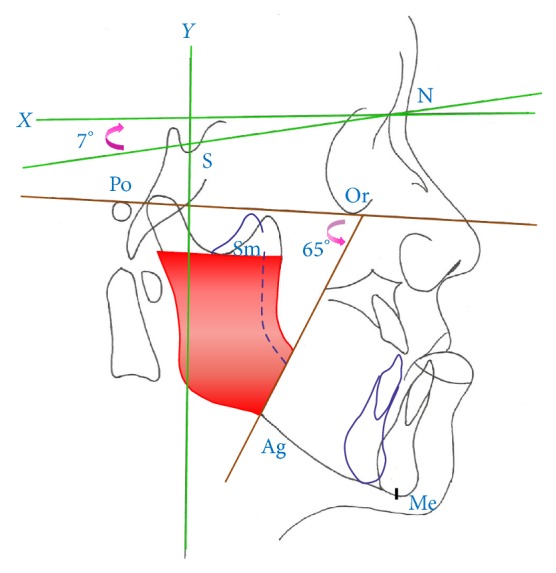
Landmarks: sella (S), nasion (N), menton (Me), antegonial notch (Ag), sigmoid notch (Sm), orbitale (Or), and porion (Po). *x*-axis (green line): constructed by drawing a line through nasion 7° up from SN line. *Y*-axis (green line): a line through sella (S) perpendicular to the *x*-axis. FH (Frankfort horizontal) line (brown color): a line connecting Po to Or. PMS (pterygomasseteric sling) line (brown color): a line through Ag point 65° to FH line. Area of PMS (red color): between PMS line and a line through Sm parallel to FH line. Blue dash line: postoperative ramus position.

**Figure 2:**
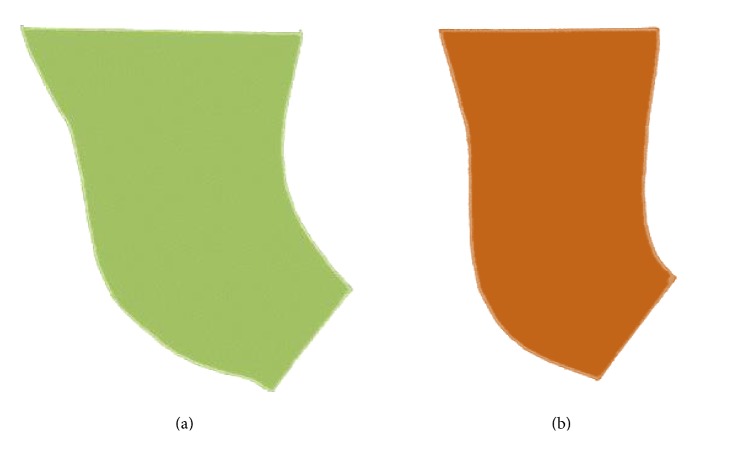
(a) Preoperative area of PMS (green color). (b) More than 1-year postoperative area of PMS (brown color).

To evaluate the postsurgical stability, serial lateral cephalograms (preoperatively [T1], immediately after surgery [T2], and more than one year postoperatively [T3]) were obtained. The landmarks were identified as follows: sella (S), nasion (N), menton (Me), and antegonial notch (Ag), and sigmoid notch (Sm). For analysis, the *x*-*y* coordinate axis was constructed. This coordinate system had its origin at the point N and its *x*-axis formed an angle of 7 degrees upward with the reference line (NS) as the horizontal axis [9]. Similar to Lee's report [10], a PMS plane was proposed at Ag point a 65° angle relative to Frankfort horizontal (FH) plane (Figure 1). The preoperation and postoperation areas of ramus were identified in the Figure 2. The surgical changes were defined as follows: postsurgical immediate change (T21), postoperative stability (T32), and the final surgical change (T31). Investigating the postoperative stability (T32), IVRO can display both horizontal movements (forward and backward). Therefore, we divided the mandibular movements of postoperative stability (T32) into 3 groups (the total movement group, forward movement group, and backward movement group).

The parameters related to the linear cephalometric measurements were identified to evaluate the mandibular position; the reference points and lines were identified on the cephalograms. Positional changes in the landmarks were noted relative to this coordinate system. Relapse was defined as the forward displacement of the Me. The area of PMS was measured using the NIH ImageJ software. The variables, surgical changes (T21), and postoperative stability (T32) were calculated and analyzed by Student's* t-*test. Pearson's correlation coefficient was used to detect the correlations between the cephalometric parameters. The multiple regression analysis was used to assess the association between risk factors and postoperative relapse. A *p* value of less than or equal to 0.05 was considered significant. The null hypothesis is that there is no significant correlation between PMS (T31) and postoperative stability (T32). This retrospective case study followed the principles of the Declaration of Helsinki and was approved by the human investigation review committee at the Kaohsiung Medical University Hospital (KMUH-IRB-20140362).

## 3. Results

**Figure 3:**
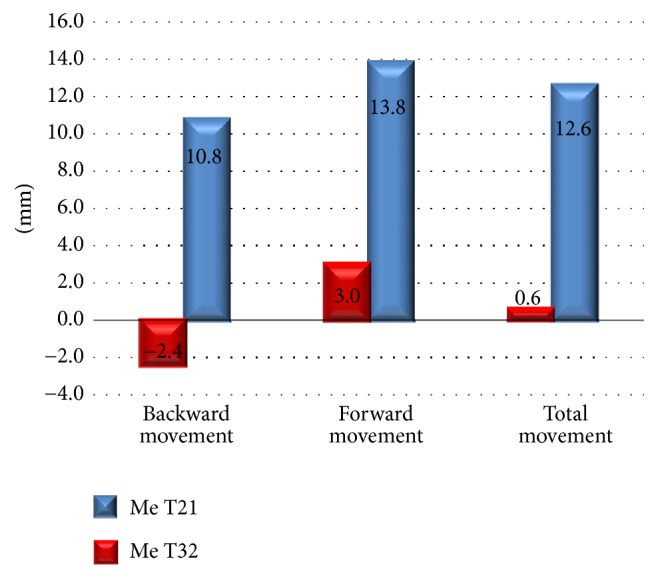
Relation between immediate surgical change Me (T21) and postoperative stability Me (T32) in all movements.

There were 26 female and 14 male patients, with a mean age of 20.5 years. The mean follow-up period for these patients was 28.5 months. The mean changes of the landmark positions in horizontal and vertical directions after surgery are summarized in Table 1. The mean setback of the Me was 12.6 mm in the horizontal direction, with 0.6 mm downward in the vertical direction. Regarding the postoperative stability of the follow-up (T32), the mean changes of the Me were 0.6 mm forward in the horizontal direction and 0.5 mm upward in the vertical direction, and reduction of PMS's area was 291 mm^2^ (17.2%). Student's test reveals significant changes in the postoperative changes (T21 and T31) and reduction of PMS's area.  Figure 3 presented the relation between Me immediate setback (T21) and postoperative stability (T32) in all movements.

**Figure 4:**
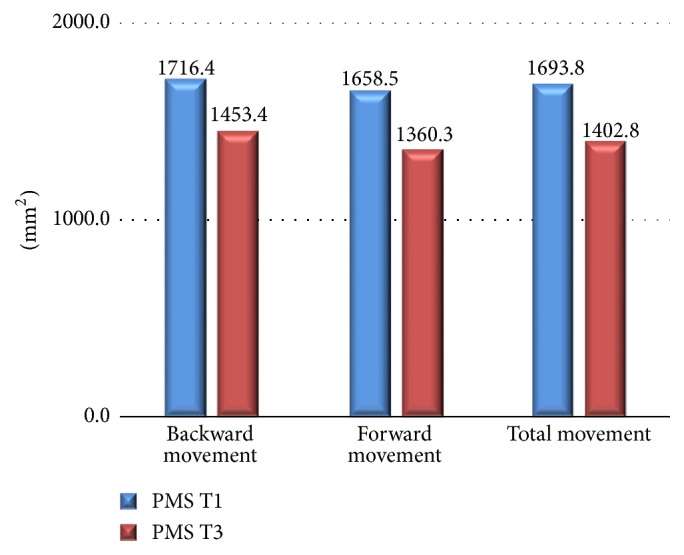
The area of PMS at T1 and T3 in all movements.

Table 2 shows Pearson's correlation coefficients (total movement group) and their significance, which were calculated between Me-T32 and the changes in all variables. Significant correlations were established between horizontal Me-T32 and the amount of setback (immediately postoperation Me-T21; long-term postoperation Me-T31) and the change in the vertical direction of Me-T21 and Me-T32. There is weak correlation between amount of setback and the changes of PMS's area. Significant correlations were established between vertical Me-T32 and the amount of setback (immediately postoperation Me-T21) and long-term relapse (Me-T31), the change in the vertical direction of Me-T21 and Me-T31, and the changes of PMS's area (T31).  Figure 4 showed both areas of PMS at T1 and T3.

**Figure 5:**
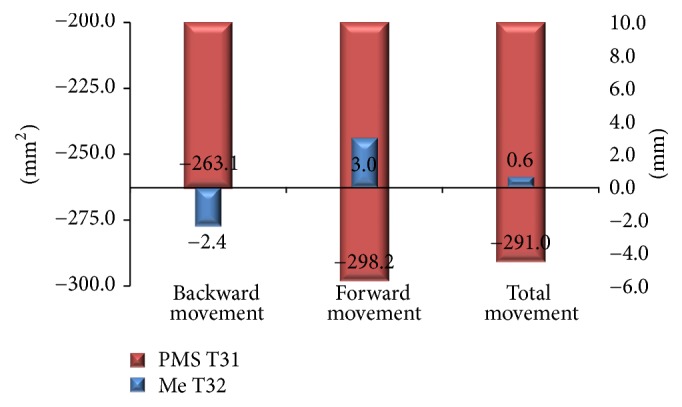
Relation between reduction area of PMS (T31) and postoperative stability of Me (T32).

Investigating the direction of horizontal movement (Table 3), 22 patients (55%) had a mean of 13.8 mm setback (T31) and 3.0 mm forward movement (T32), 17 patients (42.5%) are with a 10.8 mm setback (T31) and 2.4 mm backward movement (T32), and 1 patient (2.5%) was without movement (T32). The reduction of PMS's area (T31) was 298.2 mm^2^ (18%) and 263.1 (15.3%) mm^2^ in forward and backward groups. Comparing forward and backward groups, Student's test reveals significant changes in the horizontal changes (T21 and T32) and vertical change (T32). However, there is no significant difference in the area of PMS (T1, T3, and T31) between forward and backward groups.  Figure 5 revealed the relation between reduction area of PMS (T31) and postoperative stability of Me (T32).

Table 4 shows Pearson's correlation coefficients and their significance, which were calculated between Me-T32 and the changes in forward and backward groups. In forward group, significant correlations were established between relapse (T32) and the amount of setback (T21) and the change in the vertical direction of (T32 and T31). There is weak correlation between amount of setback and the changes of PMS's area. Significant correlations were established between vertical change (T32) and the amount of setback (T21 and T32), the change in the vertical direction (T21 and T31), and the changes of PMS's area (T31). In backward group, there is weak correlation between Me (T32) and variables (horizontal and vertical change of Me and area of PMS).

In total patients group, multiple linear regression model was significant: Horizontal Relapse (T32) = −3.008 − 0.307 (T21 setback) + 0.544 (T21 vertical change) + 0.002 (T31 PMS's area). *R*^2^ = 0.3048 (*p* < 0.05) showed the value in prediction of horizontal relapse (T32). In forward group, multiple linear regression model was significant: Horizontal Relapse (T32) = −0.421 − 0.271 (T21 setback) + 0.014 (T21 vertical change) + 0.001 (T31 PMS's area). *R*^2^ = 0.3768 (*p* < 0.05) showed the value in prediction of horizontal relapse (T32). In backward group, multiple linear regression model was not significant (*R*^2^ = 0.1514, *p* > 0.05).

## 4. Discussion

The direction and amount of facial bone growth vary among individuals; however, if the morphology and growth rate of the facial bones are affected, this may lead to abnormal bone morphology and abnormities in the maxilla and mandible and to different degrees as well as abnormal occlusion. Enlow [11] pointed out that the growth of the mandible shifts forward and downward, which produces compensating and balancing between bone apposition and resorption. Bone apposition can also be found in the bone surface at the posterior border and outer surface of the mandibular body, while resorption takes place in the anterior and inner borders of the mandibular ramus, which can be interpreted as functional remodeling and reinforcement due to the attachment and movement of muscles during the growth of the mandible. Therefore, it can be surmised that the growth and development of the mandible are affected by the varying characteristics of the different inherent tissues and their acquired function, which leads to relatively high variability in the mandibular growth. Consequently, human bone devotes its lifetime to remodeling by surrounding muscular attachment and especially after fracture or osteotomy.

As the purpose of orthognathic surgery is to correct skeletal deformity and improve chewing functions by eliminating malocclusion and restoring a balanced profile of bone proportion, the stability of the mandible is very important. In SSRO technique, Hsu et al. [12] reported 9.3 mm setback with a 1.9 mm relapse in the screw (bicortical osteosynthesis) fixation and 10.9 mm setback with a 2.7 mm relapse in the plate (monocortical osteosynthesis) fixation. The magnitudes of postoperative relapse are 20% in the screw fixation and 25% in the plate fixation. However, there is no statistically significant difference between two groups during surgery and postoperative follow-up period. In the IVRO technique, recent studies [2, 13, 14] reported that 1-year postoperative movements are backward with 0.8 mm (7.6% = 0.8/10.5), 2.3 mm (3.7% = 0.2/5.4), and 0.1 mm (1% = 0.1/12.4), respectively.

It is still controversial that magnitude of postoperative relapse is related to amount of setback, either SSRO or IVRO technique. Owing to difference in osteotomy designs between SSRO and IVRO, we found that direction and magnitudes of postoperative relapse had changed after long-term follow-up (at least 2-year). In our previous report [15], we found that mandibular movement had changed from backward (1 year) to forward (2 years). It means that only 1-year follow-up could not explore the final result of relapse in IVRO than SSRO technique. Therefore, it is very important to avoid misunderstanding on the postoperative stability or relapse in the research of IVRO. In the present study, postoperative relapse (T32) of total group was 0.6 mm (4.8% = 0.6/12.6) forward significantly. During the long-term follow-up, we should not ignore any direction of postoperative movement. According our findings (T32), 22 patients (55%) had a 3.0 mm in forward movement (3/13.8 = 21.7%) with significance and 17 patients (42.5%) had a 2.4 mm backward movement (2.4/10.8 = 22.2%) without significance, and 1 patient (2.5%) without movement. Therefore, amount of relapse (forward movement) was offset by backward movement. In the vertical direction, Me maintains stability and its total change (T31) was only 0.1 mm downward. There were significant correlations between postoperative relapse (T32) and amount of setback (T21) in the horizontal and vertical directions. Comparing to amount of setback (12.6 mm), vertical changes (T21, T32, and T31) were small and less than 1 mm in average. While evaluating risk factors of postoperative relapse, vertical change was usually treated as a minor factor, even if it was statistically significant. Comparing to forward movement (T32), backward movement (T32) presents weak correlation in Pearson's test and poor prediction in the multiple regression model (*R*^2^ = 0.1514, *p* > 0.05). In forward group, relapse was significant correlation to amount of setback. In forward group, multiple linear regression model (*R*^2^ = 0.3768, *p* < 0.05) showed the value in prediction of horizontal relapse (T32).

In particular, without fixation between the proximal and distal segments by IVRO, the PMS's area of the PMS may be an important factor related to postoperative mandibular stability. A setback of the mandible may also stretch the PMS. Investigating the postoperative patterns of ramus, anterior-posterior dimension of ramus was significantly narrowed by mandibular setback using IVRO. Comparing to IVRO, ramus was split by SSRO and its anterior-posterior dimension presented little change. IVRO caused the greater potency and variety of bone remodeling due to no fixation between proximal and distal segments. The remodeling process of IVRO could presume to be affected by PMS compared to SSRO which was fixation between proximal and distal segments. The PMS is made up of masseteric and medial pterygoid muscles. The medial pterygoid muscle, which originates from the pterygoid fossa of the sphenoid bone and tuberosity of the maxilla, is inserted on the medial surface of the mandible. Its fibers pass downward, laterally, and posteriorly and are inserted, by a strong tendinous lamina, into the lower and back part of the medial surface of the ramus and angle of the mandible, as high as the mandibular foramen. Different from SSRO, sigmoid notch had been osteotomies through by IVRO. Therefore, superior portion of PMS affects the ramus area which could be as high as near to sigmoid notch except condylar and coronoid processes. The origin of the masseter muscle is on the lateral part of the zygomatic arch and is inserted into the angle of the mandible. Consequently, inferior portion of PMS was proposed at Ag point a 65° to FH plane. Hence area of ramus change could be detected.

Image J is a public domain Java image processing software that draws its inspiration from NIH. It is capable of supporting commonly used image processing functions, like contrast manipulation, sharpening, smoothing, edge detection, and median filtering. Realistic dimensional measurements in units like millimeters can be provided by spatial calibration. By way of defined selections and intensity threshold, Image J can calculate its area and pixel value statistics. Therefore, Image J is good tool for us that is fulfilled to investigate image processing of PMS's area and analysis.

The capacity of the PMS was affected by the osteotomy position. The position of the vertical osteotomy site in our method should be close to the posterior border of the mandibular foramen, while the osteotomy lines at the anterior and inferior positions of the mandibular foramen should be cut forward as far as possible; that is, the osteotomy line should be set upwards to the coronoid process and downwards to the direction of the mandibular body. In our opinion, the position of osteotomy is directly correlated with the endurable capacity of the PMS. In other words, the accommodate capacity of the PMS is bigger when the osteotomy is positioned forward. In our study, correlation analyses were performed to evaluate the relationship between the reduced area of PMS and relapse at point Me in the total patients. However, Pearson's correlation coefficient revealed no significance. Even when the reduced area of the PMS was significant, it still was not significant in relation to postoperative relapse. Therefore, a significant reduction in the area of the ramus did not affect the maintenance of postoperative stability. Even opposite direction of movement, original area, and its reduction value were similar between forward and backward group.

Postoperative mandibular stability is determined by not only surgery but also the preparation of orthodontic treatment. A postoperative stable occlusion can reduce the shifting of teeth and may be helpful in the prevention of relapse. If preoperative orthodontic treatment cannot provide a postoperative stable occlusion, patients experience shifting or protrusion of the mandible to adapt to the interference in occlusion and find better occlusion sites after releasing the intermaxillary fixation, and such improper movements may lead to relapse after operation. Therefore, orthodontists and surgeons must establish excellent communication with one another.

Although there is much remaining to be done, our study revealed that postoperative relapse was complex and affected by multiple factors. In conclusion, amount of setback was significantly related to relapse and the reduced area of the PMS was not. However, the limitations of the present study are lack of three-dimensional (3D) pterygomasseteric sling volume and bilateral setback amounts of mandible. In the further research, the 3D analysis can enable us to study musculoskeletal changes after orthognathic surgery.

## Figures and Tables

**Table 1:** Student's *t*-test for significance for the menton and area of PMS (total group; *n* = 40) among T1, T2, and T3.

Variable	Mean	SD	*p* value
Horizontal change (mm)			
T21	−12.6	4.34	<0.001^*∗*^
T32	0.6	3.17	0.225
T31	−11.9	4.02	<0.001^*∗*^
Vertical change (mm)			
T21	0.6	1.85	0.059
T32	−0.5	1.80	0.098
T31	0.1	1.65	0.743
Area of PMS (mm^2^)			
T1	1693.8	237.77	
T3	1402.8	231.89	
T31	−291.0	149.37	<0.001^*∗*^

*n*: number of patients; PMS: pterygomasseteric sling; ^*∗*^significant *p* < 0.05; T21: immediate surgical change; T32: postoperative stability; T31: final surgical change.

**Table 2:** Pearson correlation testing for the skeletal postoperative stability (T32) at the menton (total group; *n* = 40).

Variable	T32	
	Horizontal	Vertical
Horizontal change (mm)		
T21	−0.463^**∗**^	0.394^**∗**^
T32	1	−0.589^**∗**^
T31	0.288^**∗**^	−0.039
Vertical change (mm)		
T21	0.386^**∗**^	−0.589^**∗**^
T32	−0.589^**∗**^	1
T31	−0.208	0.428^**∗**^
Area of PMS (mm^2^)		
T1	−0.006	−0.080
T3	−0.098	0.204
T31	−0.143	0.445^**∗**^

*n*: number of patients; PMS: pterygomasseteric sling; ^**∗**^significant *p* < 0.05; T21: immediate surgical change; T32: postoperative stability; T31: final surgical change.

**Table 3:** Student's *t*-test for significance for T32 menton between forward movement group (*n* = 22) and backward movement group (*n* = 17) among T1, T2, and T3 periods.

Variable	Forward		Backward		*p* value
	Mean	SD	Mean	SD	
Horizontal change (mm)					
T21	−13.8	4.41	−10.8	3.93	0.034^**∗**^
T32	3.0	1.89	−2.4	1.78	<0.001^**∗**^
T31	−10.8	3.59	−13.2	4.37	0.072
Vertical change (mm)					
T21	1.0	1.71	−0.03	2.01	0.086
T32	−1.2	1.76	0.5	1.52	0.003^**∗**^
T31	−0.2	1.37	0.4	2.04	0.261
Area of PMS (mm^2^)					
T1	1658.5	168.38	1716.4	298.11	0.447
T3	1360.3	161.70	1453.4	302.00	0.224
T31	−298.2	102.60	−263.1	182.93	0.451

*n*: number of patients; no movement (*n* = 1); PMS: pterygomasseteric sling; ^**∗**^significant *p* < 0.05; T21: immediate surgical change; T32: postoperative stability; T31: final surgical change.

**Table 4:** Pearson correlation testing for T32 forward movement group (*n* = 22) and backward group movement (*n* = 17).

Variable	Forward (T32)		Backward (T32)	
	Horizontal	Vertical	Horizontal	Vertical
Horizontal change (mm)				
T21	−0.611^**∗**^	0.427^**∗**^	0.037	0.033
T32	1	−0.552^**∗**^	1	−0.224
T31	−0.226	0.235	0.440	−0.062
Vertical change (mm)				
T21	0.214	−0.690^**∗**^	0.385	−0.359
T32	−0.552^**∗**^	1	−0.224	1
T31	−0.445^**∗**^	0.428^**∗**^	0.213	0.389
Area of PMS (mm^2^)				
T1	0.032	−0.354	0.334	0.018
T3	−0.097	0.008	0.303	0.254
T31	−0.205	0.593^**∗**^	−0.043	0.390

*n*: number of patients; no movement (*n* = 1); PMS: pterygomasseteric sling;  ^**∗**^significant *p* < 0.05; T21: immediate surgical change; T32: postoperative stability; T31: final surgical change.
